# Genomic epidemiological analysis of a single-centre polyclonal outbreak of *Serratia marcescens*, Belgium, 2022 to 2023

**DOI:** 10.2807/1560-7917.ES.2024.29.48.2400144

**Published:** 2024-11-28

**Authors:** Sam Van Goethem, Basil Britto Xavier, Youri Glupczynski, Matilda Berkell, Philippe Willems, Bruno Van Herendael, Katrien Hoet, Katleen Plaskie, Daan Van Brusselen, Herman Goossens, Surbhi Malhotra-Kumar

**Affiliations:** 1 Laboratory of Medical Microbiology, Vaccine and Infectious Diseases Institute, University of Antwerp, Antwerp, Belgium; 2 ZAS Hospital Network, Antwerp, Belgium; 3 Multidisciplinary Unit of Infectious Diseases, GZA (ZAS) Hospitals, Antwerp, Belgium; 4 Laboratory of Medical Microbiology, GZA (ZAS) Hospitals, Antwerp, Belgium; 5 Department of Neonatology, GZA (ZAS) Hospitals, Antwerp, Belgium; 6 Department of Paediatrics, GZA (ZAS) Hospitals, Antwerp, Belgium; 7 Antwerp Paediatric Clinical Trial Network on vaccines and infectiology, University of Antwerp, Antwerp, Belgium

**Keywords:** *Serratia marcescens*, NICU, outbreak, whole genome sequencing

## Abstract

*Serratia marcescens* is an opportunistic pathogen with a propensity to cause nosocomial outbreaks, particularly in neonatal intensive care units (NICUs). We present a sustained outbreak spanning over 18 months (1 January 2022–29 August 2023) in a NICU in Antwerp, Belgium, affecting 61 neonates, identified through samples taken for diagnostic purposes and by rectal screening. Ten neonates were infected: five with lower respiratory tract infection, four with conjunctivitis and one fatal case with sepsis. In a logistic regression analysis, nursing in an incubator was significantly associated with acquisition of *S. marcescens* (odds ratio (OR): 2.99; 95% confidence interval (CI): 1.14–8.25; p < 0.05). Whole genome sequencing-based multilocus sequence typing (wgMLST) and core genome single nucleotide polymorphism (cgSNP) analysis of isolates from clinical (n = 4), screening (n = 52) and environmental samples (n = 8), identified eight clusters and five singletons not associated with the clusters. Although outbreak measures were successful in containing further spread within the ward during sudden surges when > 4 cases per week were identified (peak events), several peaks with different clonal clusters occurred. The emergence of similar outbreaks in Belgian hospitals underscores the need of continuous surveillance and NICU-specific infection prevention and control (IPC) measures.

**Key public health message**

**What did you want to address in this study and why?**

*Serratia marcescens* is a bacterium found in a large variety of environments, from soil and water to insect guts or hospital wards. It is a frequent cause of outbreaks in neonatal intensive care units (NICU). We investigated an outbreak with *S. marcescens* in a Belgian NICU to identify the transmission sources, assess the risk factors for infection and evaluate the effectiveness of implemented infection prevention strategies to contain the outbreak.

**What have we learnt from this study?**

We detected *S. marcescens* from 61 newborns and 71 environmental samples from the NICU. One case died. The bacterium was more often detected from newborns nursed in an incubator. Several variants of the bacterium were found in the patient and in the environmental isolates. We could not identify the source of the outbreak.

**What are the implications of your findings for public health?**

Control measures including intensive screening of patient and the environment, patient isolation and enhanced cleaning prevented further spread within the department after an increase of cases but did not prevent a new surge of cases. As these outbreaks can have severe consequences, continuous surveillance of neonates and the inanimate environment should be introduced in the NICUs.

## Introduction

*Serratia marcescens,* the most important opportunistic human pathogen in the *Serratia* genus, often causes outbreaks of hospital-associated infections, particularly in neonatal intensive care units (NICUs) [1-3]. The incidence of late-onset neonatal sepsis caused by *S. marcescens* has been estimated to 2.3 infections per 1,000 preterm infants. The infection is associated with significant morbidity and a reduced rate of survival (adjusted relative risk (RR): 0.88) [4]. Risk factors among neonates for acquisition of or infection with *Serratia* are low birth weight (< 1,500 g), use of broad-spectrum antimicrobials, complex chronic conditions and indwelling catheter lines [3,5,6]. Outbreaks of *S. marcescens* are challenging to control and require early detection and rapid implementation of strict infection prevention and control (IPC) measures [7]. Although environmental sources of infection can usually not be identified and cross-transmission via hands of healthcare workers is assumed to be the main mode of transmission, water sources (e.g. washbasins, air conditioning), liquid nutrition (e.g. breast milk, baby formula, total parenteral nutrition), soaps, disinfectants and medication have been implicated in *Serratia* outbreaks [3,8,9].

In Belgium, surveillance of infectious diseases is performed by the Scientific Institute of Public Health, Sciensano, with support by the National Reference Centers. No surveillance is performed for infectious diseases in neonatal (intensive care) units.

## Outbreak detection

**Figure 1:**
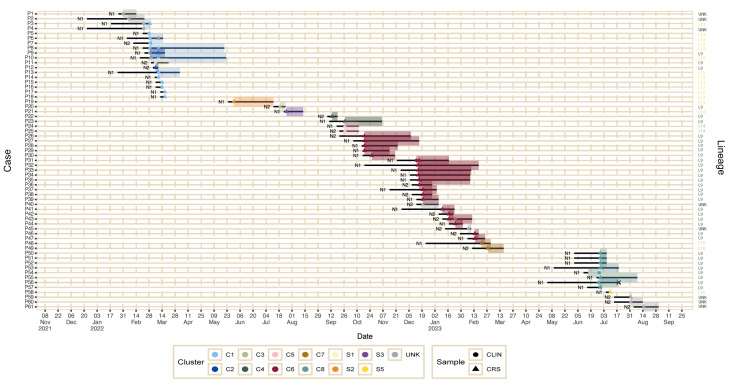
Timeline of detection of cases with *Serratia marcescens* in an outbreak in a neonatal intensive care unit, Belgium, 2022–2023 (n = 61)

The index case (P5) was detected in the end of February 2022 when a preterm neonate born at 26 weeks of gestation rapidly developed a fatal septic shock, and *S. marcescens* was detected from blood cultures (Figure 1). Contemporary detection of *S. marcescens* from a clinical specimen, a conjunctival swab, from another neonate (P4) in the NICU (Figure 1) led us to perform a retrospective review of the laboratory information system database. This revealed that *S. marcescens* had been identified from various clinical specimens from three other NICU patients (P1–P3) (Figure 1) in the preceding 3 weeks. In contrast, *S. marcescens* had been detected in only one neonate hospitalised in the NICU during 2021. An outbreak was declared when rectal screening of the neonates in NICU in early March identified three colonised patients (P6–P8) and one additional patient with conjunctivitis (P9).

C: cluster; CLIN: detection of *S. marcescens* from a clinical specimen; CRS: screening sample; L: lineage; N1: intensive care; N2: medium care; P: patient; *S*: *Serratia*; S: singleton cluster; UNK: unknown cluster type, *S. marcescens* isolate not sequenced.

In the graph, start and end dates of hospitalisation stay of all identified cases are presented (P1–P61). Hospital stay is indicated by horizontal lines, where different colour-markings indicate the *S. marcescens* strain cluster.

In this report, we describe a prolonged *S. marcescens* outbreak in a NICU in Belgium and the impact of sequencing analysis on the understanding of strain epidemiology throughout the outbreak period.

## Methods

### Outbreak setting

The outbreak occurred in a 580-bed secondary care hospital, a member of the GZA hospital network that houses the largest maternity services in Antwerp with > 5,000 births recorded annually. The NICU has 27 beds separated into two physically distinct areas: the intensive care area (N1, 15 incubators) for very premature (< 32 weeks of gestational age) or critically ill neonates and the medium care area (N2, 12 cots) for neonates in less critical condition, as presented in Supplementary Figure 1. During periods with no outbreaks, staff and medical equipment (diaper scales, temperature probes, monitors etc.) are shared between the two areas. Within the NICU, one common area (N) is used as an entrance with a desk space and several washbasins for parents and staff to wash their hands before entering the NICU, as presented in Supplementary Figure 1.

### Case definitions

A case was defined as any neonate hospitalised in the NICU and colonised or infected with *S. marcescens* between 1 January 2022 and 29 August 2023. Infected cases were defined as patients with a *S. marcescens*-associated infection according to the diagnostic criteria by European Centre for Disease Prevention and Control (ECDC) [10]. All other cases were considered as colonised cases. Peak events were defined as time periods in which ≥ 4 new cases were identified in a week. The attack rate was defined as the cumulative incidence proportion over a specified period.

### Environmental sampling of the neonatal intensive care unit

Drains (washbasins, baths) in different parts of the NICU were sampled twice weekly. Additionally, other environmental samples (feeding tubes, keyboards, water taps, outer surfaces of neonatal incubators, diaper scales, breast pumps, eye drops, liquid soaps, parenteral nutrition and prepared formula milk) were taken on 2 March 2022 and on 23 December 2022 to identify possible sources of the outbreak.

### Microbiological methods

Rectal swabs, obtained once a week from all patients admitted to the NICU, were plated onto a chromogenic agar specific for *Serratia* spp. (Serratia Colorex, bioTrading, Mijdrecht, the Netherlands) and incubated at 37°C overnight. Detection of *S. marcescens* from clinical specimens was performed using standard bacteriological procedures according to the Clinical Microbiology Procedures Handbook [11]. Environmental samples were obtained by swabbing surfaces using eSwab (Copan, Brescia, Italy). Isolates of *S. marcescens* were identified using MALDI-TOF (Bruker, Billerica, the United States (US)). Susceptibility testing was performed by disk diffusion according to the European Committee on Antimicrobial Susceptibility Testing (EUCAST) criteria (version 13.1) [12].

### Whole genome sequencing

All *S. marcescens* isolates were stored at −80°C and analysed with whole genome sequencing (WGS) in four batches, on 1 September 2022, 25 October 2022, 2 January 2023 and 21 August 2023. Genomic DNA was extracted using MasterPure Complete DNA kit (LGC Biosearch Technologies, Hoddesdon, the United Kingdom (UK)). Multiplexed Nextera XT libraries were prepared and sequenced using 2 × 250 bp paired-end sequencing on a MiSeq instrument (V2 500 cycles, Illumina Inc., San Diego, US). Trimming (Trim Galore [13] version 0.6.6), assembly (SPAdes [14] version 3.13.1) and annotation (prokka [15] version 1.12) was performed using BacPipe version 1.2.6 [16]. Whole genome based multilocus sequence typing (wgMLST) was performed using a gene-by-gene approach-based allelic loci comparison (chewBBACA [17] version 2.5.5) by generating a customised study-specific scheme. The wgMLST allelic loci distances were calculated (chewBBACA), and the generated MSTree visualised (GrapeTree [18] version 1.5.0). Nine reference strains, shown in Supplementary Table 1, were used to assign *S. marcescens* lineages 9–16 as defined by Williams et al. [1]. A core genome single nucleotide polymorphism (cgSNP) alignment was generated (parSNP [19] version 1.7.4) and used to construct a maximum-likelihood tree (IQ-TREE [20] version 2.0.6) and visualised (ggtree in R version 2022.07.2 [21]).

### Analytical epidemiological investigations and statistical analysis

In a retrospective case–control study, we compared cases detected between 1 March 2022 and 1 January 2023 with patients with no *S. marcescens* detected (infection or colonisation). The controls were selected from time periods with a higher incidence of cases: 1–17 March 2022, 8–23 October2022 and 13–28 December 2022. These time periods were selected to avoid having a control group of patients when there was either no or low recorded presence of *S. marcescens*. We included variables nursing in an incubator, gestational age and length of stay.

A test for multicollinearity was performed with the selected variables (olsrr-package in R, https://www.r-project.org/). Multiple logistic regression was performed for nursing in an incubator and a lower mean gestational age at birth (glm, stats-package in R). A p value of < 0.05 was considered statistically significant.

## Results

### Descriptive epidemiology

**Figure 2:**
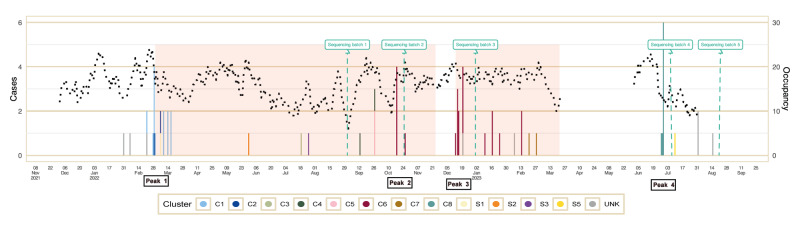
Epicurve of cases with *Serratia marcescens* and control measures taken in an outbreak in a neonatal intensive care unit, Belgium, 2022–2023 (n = 61)

In total, we identified 61 cases between 1 January 2022 and 29 August 2023. An overview of cases is presented in the epicurve within Figure 2, including a timeline for each individual case. Fifty neonates were identified as a case by rectal screening only. Four cases (P1–4) were retrospectively detected between 1 January and 1 March 2022, P3 also had a positive rectal screening early March. Ten neonates presented with a clinical infection caused by *S. marcescens*: five with lower respiratory tract infection, four with acute conjunctivitis and one with bloodstream infection. The index case died from the infection; the other cases survived. All isolates had a wild-type antimicrobial susceptibility pattern for amoxicillin, amoxicillin-clavulanic acid, piperacillin-tazobactam, cefuroxime, ceftriaxone, ceftazidime, cefepime, meropenem, amikacin, tobramycin, ciprofloxacin and sulfamethoxazole-trimethoprim and none showed acquired resistance. During peak events (1–17 March 2022, 18 October 2022, 13–20 December 2022, 27–29 June 2023), attack rates varied between 10 of 27 patients (13–20 December 2022) and 16 of 32 patients (1–17 March 2022).

C: cluster; *S*: *Serratia*; S: singleton cluster; UNK: unknown cluster type, *S. marcescens* isolate not sequenced.

Time of sequencing and start and stop of outbreak measures (red boxes) are indicated. Total occupancy in the ward is represented by a dotted line on the epicurve. Following outbreak control and response measures were taken during the marked time periods (red boxes): systematic rectal screening once a week, contact isolation (cohort), dedicated nursing, environmental screening (washbasin drains) and enhanced cleaning and disinfection.

### Environmental investigations

**Figure 3:**
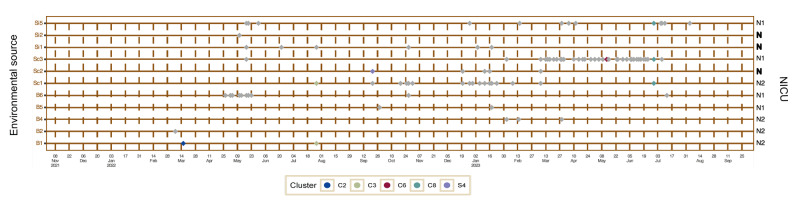
Timeline of detection of *Serratia marcescens* from drain samples in an outbreak in a neonatal intensive care unit, Belgium, 2022–2023 (n = 1,380)^a^

*Serratia marcescens* was detected from 71 of approximately 1,380 drain swab samples but not from any other environmental samples (n = 32) (Figure 3)*.* Of the 16 drains in the NICU, *S. marcescens* was isolated from 11 of them (N1: 4/7, N2: 4/5, common space: 3/4) (Figure 3). When considering positivity per week, the positivity ratio of all screened drains was 5.8% (71/1,224).

B: bath drain; C: cluster; N: NICU common space between N1 and N2; N1: intensive care; N2: medium care; *S*: *Serratia*; S: singleton cluster; Sc: drain of a large washbasin; Si: drain of a small washbasin.

^a^ The number of drain samples taken is an approximation. *Serratia marcescens* was detected from 71 drain samples.

### Whole genome sequencing analysis

**Figure 4:**
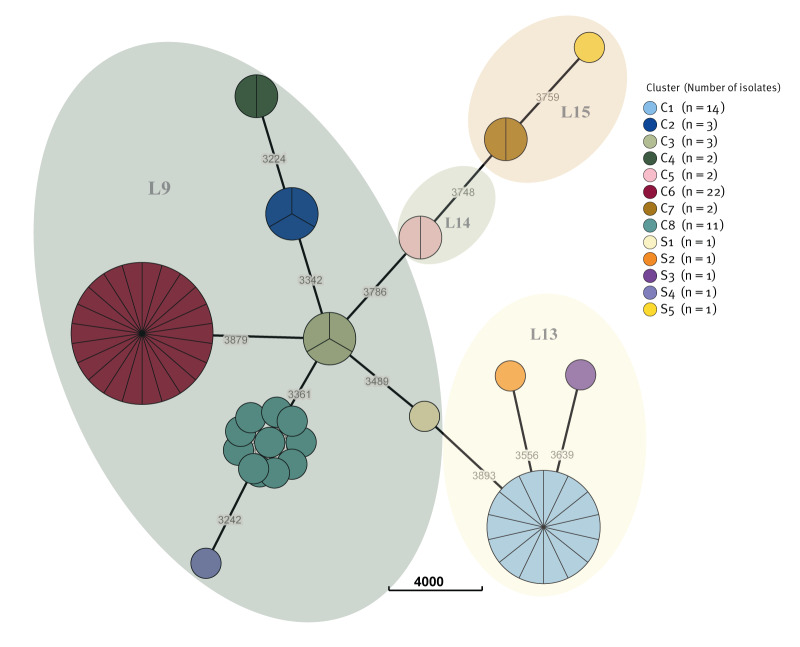
Minimal spanning tree of *Serratia marcescens* isolates in an outbreak in a neonatal intensive care unit, Belgium, 2022–2023 (n = 64)

We sequenced 56 *S. marcescens* isolates retrieved from the 61 cases. The isolates were from rectal swabs (n = 51), blood culture (n = 1), endotracheal aspirates (n = 2), sputum (n = 1) and conjunctival swab (n = 1). Eight isolates from the drain swabs were sequenced: bath1 (B1, n = 2), washbasin1 (n = 2), washbasin2 (n = 1), washbasin3 (n = 2) and washbasin5 (n = 1). We identified eight distinct clusters with at least four isolates of *S. marcescens* (Figure 4), where each cluster was separated by > 3,200 allelic loci differences. Remarkably during peak events, two or more patients developed clinical infection due to *S. marcescens* belonging to the predominant clonal clusters. In contrast, isolates collected during time periods between peak events resulted in smaller clusters with only two isolates or singletons and did not involve patients presenting with clinical *S. marcescens* infections.

Cluster and singleton names are based on the isolation date. Numbers represent distances in allelic loci from a total of 5,033 identified loci. Clusters are collapsed to pie charts when there were < 12 loci difference between the isolates. Lineage L9 is a hospital-associated lineage, while lineages 13–15 are mostly related to water, plants and insects [1].

**Figure 5:**
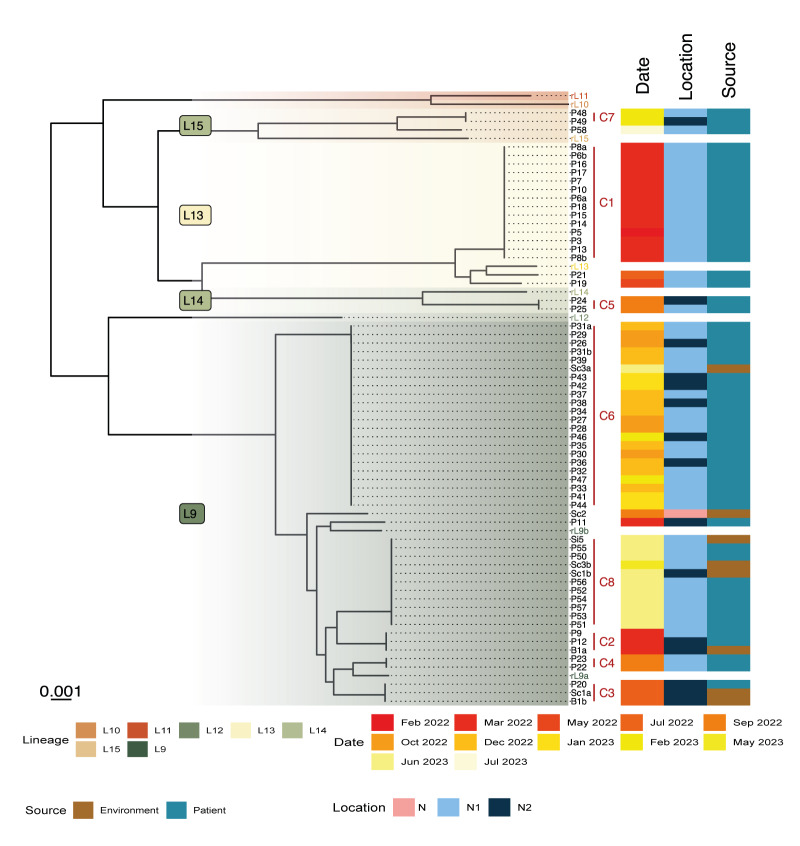
Phylogenetic tree based on core genome single nucleotide polymorphism (SNP) analysis of *Serratia marcescens* isolates in an outbreak in a neonatal invasive care unit (n = 64) and reference strains (n = 9), Belgium, 2022–2023

Further, cgSNP analysis revealed that the analysed *S. marcescens* isolates belonged to separate lineages, pointing to a broad diversity (Figure 5). Within-lineage distance was less than 7,500 SNPs, except for P48, P49 and P58, which differed by less than 5,000 SNPs with each other, but differed by 12,919 SNPs from the closest reference strain (rL15). Reference strains are listed in the Supplementary material. Between lineages, there were a minimum of 14,000 SNPs. The highest SNP distance was eight SNPs (C8), but most clusters had no SNP difference (C3–C7). Cluster C1 had a maximum of one SNP distance and C2 had two SNPs. The distance matrix is presented in Supplementary Table 2.

B: bath; C: cluster; N: common space between N1 and N2; N1: intensive care; N2: medium care; rL: reference strains of *Serratia marcescens*; Sc: large washbasin; Si: small washbasin.

Heatmaps of sampling date, sampling site and sampling source are shown. Lineages with patient strains are labelled within the tree. The tree is rooted with a reference of lineage 16 (not depicted). For case isolates, a and b represent different isolates from the same patient or site. Two reference strains for lineage 9 were used (rL9a, GCF_001294565.1_ASM129456v1 and rL9b, GCF_000739215.1_ASM73921v1) to include a broader coverage of this lineage.

A total of 34 sequenced patient isolates and all eight isolates from the drains belonged to a hospital-associated *S. marcescens* lineage (lineage 9) [1]. All the other isolates, including those isolated from the initial outbreak cluster (C1), belonged to lineages not associated with hospitals (lineage 13–15).

Sequencing of the isolates demonstrated concurrent presence of *S. marcescens* in both patients and drains in four of five distinct clonal clusters: C2 on 15 March 2022, C3 on 27 July 2022, C6 on 13 May 2023 and C8 on 29 June 2023 (Figure 1, Figure 3). *Serratia marcescens* isolated from the drain of a large washbasin, washbasin2 (S4), on 21 September 2022, did not belong to any cluster. On 13 May 2023, *S. marcescens* (Sc3a, C6) was isolated from a large washbasin, washbasin3, 2.5 months after the discharge of the last known patient (P47) with an isolate belonging to this cluster (Figure 1).

### Analytical epidemiology

During peak events with larger clonal clusters, most cases were nursed in N1 where all infants are in incubators. Length of stay was excluded from the regression analysis as the calculated tolerance was < 0.6. Nursing in an incubator was associated with detection of *S. marcescens* (odds ratio (OR): 2.99; 95% confidence interval (CI): 1.14–8.25; p < 0.05) (Table). An illustration of the NICU can be seen in Supplementary Figure 1.

**Table:** Number of cases and controls in an investigation of an outbreak of *Serratia marcescens* in a neonatal intensive care unit, Belgium, 2022–2023^a^

Variables	Cases n = 49	Controls n = 44	OR	95% CI	p value
Nursing in an incubator	39	24	2.99	1.14–8.25	0.04
Mean gestational age at birth	31 weeks 6 days	34 weeks 1 day	0.98	0.95–1.00	0.03
Mean length of stay	37.7 days	29.4 days	0.99	0.97–1.01	0.4

CI: confidence interval; OR: odds ratio.

^a^ Cases were patients with *S. marcescens* and hospitalised 1 March 2022–1 January 2023. Controls were patients with no *S. marcescens* detected and hospitalised during time periods with higher case incidence: 1–17 March 2022, 8–23 October 2022 and 13–28 December 2022.

## Outbreak control measures

After the detection of the outbreak, a multidisciplinary outbreak control team was assembled consisting of the heads of departments (physician and nurse), a microbiologist, a paediatric infectious disease specialist, infection control and prevention specialists and a representative of the board of directors of the hospital. At the start of the outbreak, we decided not to close the ward but to limit new admissions, initiate contact isolation measures by rearranging the location of the cases and labelling a case visible for staff but not for the parents or other visitors and start systematic rectal screening of neonates in the ward (Figure 2). The limitation on new admissions was implemented, as during the outbreak, the occupancy in the ward was high which could have had a limiting effect on the correct implementation of IPC measures. As a full closure would have a considerable impact on the activity of the maternity centre of our hospital and the surrounding hospitals, we decided to limit new admissions than force a full closure. During the first week of the outbreak declaration, daily on-site visits by the outbreak control team took place to investigate possible outbreak sources and transmission routes. Dedicated nursing staff was assigned and cohorting of the affected neonates was initiated. Cleaning and disinfection practices within the ward were reinforced by assigning specialised personnel. Following the initial meeting on 1 March 2022, in which outbreak control measures were set, weekly team meetings were held to re-evaluate the situation. The admission limitation was discontinued on 31 March 2022 once a case-free interval of 2 weeks was observed and after 13 cases had been discharged from the ward.

After the first sequencing batch was analysed on 1 September 2022, which revealed polyclonality of the outbreak and that origin from a single environmental source was unlikely, we decided to adapt our guidelines aiming to terminate all measures after a case-free interval of 2 weeks, including rectal screening. We decided to continue the systematic screening of drains. When a new case was detected on 13 February 2023, the previously applied outbreak control measures were re-introduced, except for restriction on admissions to the ward, which was not reinstated due to its significant impact on the activities of the maternity services of the hospital. Also, a new protocol for incubator disinfection was introduced as from this date, in which the incubator of a discharged neonate was disinfected with nebulised hydrogen peroxide before a new admission.

On 1 March 2023, four heated drains were installed (large washbasins: washbasin1, 2 and 3 and a small washbasin: washbasin5). These drains have a once daily thermo-disinfection protocol wherein the drain is heated to 85°C for 5–6 cycles. However, *S. marcescens* was repeatedly isolated from samples from an incorrectly installed heated drain. As a precautionary measure, all four heated drains were removed on 18 July 2023.

All outbreak measures, including rectal screening and sampling of drains were discontinued on 29 August 2023. It was decided that moving forward, all measures (disinfection by specialised personnel, contact isolation, nebulisation of the incubators, rectal screening, environmental screening) would be reinstated if *S. marcescens* was detected from clinical samples of two patients within a 2-week period.

## Discussion

During this extended outbreak, various outbreak control measures were implemented to decrease and contain the high rate of cases. As many NICUs, including ours, are open wards, an effective contact isolation can be difficult. By rearranging the location of cases and using all available spaces, in combination with a label of a case, an effective contact isolation could be achieved. However, this implicitly requires an extensive case-finding strategy. We chose to use rectal swabs as these have been shown to be the most sensitive sample and detection site [22]. Other sample types commonly employed are conjunctival swabs, samples from the respiratory tract (mainly nasal) and swabs of the umbilicus [22,23]. We preferred to have an extensive screening using only rectal swabs rather than samples from multiple sites from a single patient.

Given that *S. marcescens* was detected from only washbasin drains and not from other environmental samples during the initial environmental screening, we implemented a systematic screening of all drains on the ward. We observed that the number of positive drains reflected the number of cases in the ward. Our data and other recent studies argue for the removal of drains from NICU patient rooms and favour water-free care for neonates that could reduce NICU patient colonisation rates with Gram-negative bacteria like *S. marcescens* [24-26]. Eventually, we opted to remove most washbasins, which until now, proved effective in avoiding a resurgence.

Intensive screening of patients and hospital environment can be costly and labour intensive for the hospital and the microbiology laboratory. A continuous monitoring outside of an outbreak setting could enable an early detection of a silent spread within a ward and possibly predict the outbreak potential of a genetically well-characterised pathogen. Such a continuous monitoring might be feasible using a targeted approach i.e., tracking strains from clinical samples and a periodic screening of the environment, of which washbasin drains appear to have the most reservoir potential, and thus creating a view on the NICU pathobiome.

In a previous NICU outbreak of *Enterobacter* spp., incubators were one of the major contributing factors in transmission [27]. We found several clusters and different incubators involved in the present outbreak. However, the setup of incubators in N1 and cots in N2 limited our ability to effectively define direct causation between being nursed in an incubator and *S. marcescens* colonisation. In N2, isolates from cases colonised with *S. marcescens* belonged to the same clusters as environmental isolates and case isolates from N1, but cases in N2 were fewer than in N1. Although we also found an association between *S. marcescens* and nursing inside an incubator, we do not consider this to be the primary source of the outbreak, but rather a facilitator of rapid transmission. The use of invasive procedures as the real risk factors of colonisation also could not be ruled out [6].

Several distinct clonal clusters of *S. marcescens* belonging to four different lineages could be identified using WGS. Most of the sequenced *S. marcescens* patient isolates and all environmental isolates belonged to lineage 9 which is the major human, hospital-associated lineage. The shift of the specific clusters during the outbreak period might be linked to the control measures taken, as metabolic pathways differ between different *S. marcescens* lineages and even within lineages [1]. As a result, the use of certain antiseptics could select for specific lineages or clonal clusters. Alternatively, increased pressure on medical staff due to higher bed occupancy rates, which tend to reduce hygiene compliance, or other unmeasured factors related to the total disease burden in the ward might also have contributed to the propagation of certain strains present in the ward at that time, of which some may have had a greater potential to spread within a NICU environment [28].

Polyclonal outbreaks with *S. marcescens* are not rare in NICUs, and the source of these outbreaks often remains unclear [28-30]. To effectively prevent a resurgence or avoid an outbreak all together, will require a better understanding of the *S. marcescens* biology, its niches and its metabolic potential.

## Conclusion

The outbreak measures outlined in this report were effective in controlling the surge of *S. marcescens* of the protracted *S. marcescens* outbreak in our NICU. Based on our experience, we consider that a systematic, continuous epidemiological surveillance of neonates admitted in NICUs, as well as of the inanimate environment should be introduced to inform on various possible sources and transmission pathways of environmentally transmitted pathogens like *Serratia* during non-outbreak periods. Given that more hospitals in Belgium have recently reported similar *S. marcescens* outbreaks in NICUs, we see a value in starting a structured surveillance system at national level, as none currently exists in Belgium.

## Supplementary Data

232000 Supplementary Material
