# Supplementary material for: Genomic epidemiological analysis of a single-centre polyclonal outbreak of Serratia marcescens, Belgium, 2022 to 2023
Source: Euro Surveill. 2024 Nov 28;29(48):2400144. doi: 10.2807/1560-7917.ES.2024.29.48.2400144 (PMC11605797; doi:10.2807/1560-7917.ES.2024.29.48.2400144)

This supplementary material is hosted by *Eurosurveillance* as supporting information alongside the article 'Genomic epidemiological analysis of a single-centre polyclonal outbreak of Serratia marcescens, Belgium, 2022 to 2023', on behalf of the authors, who remain responsible for the accuracy and appropriateness of the content. The same standards for ethics, copyright, attributions and permissions as for the article apply. Supplements are not edited by *Eurosurveillance* and the journal is not responsible for the maintenance of any links or email addresses provided therein.

Supplementary materials

Supplementary Table 1. Utilized *S. marcescens* reference strains within lineages 9-16 as defined by Williams *et al.* [1]

Genbank reference	Lineage
GCF_001294565.1_ASM129456v1	L9 (a)
GCF_000739215.1_ASM73921v1	L9 (b)
GCF_000292365.1_ASM29236v1	L10
GCF_001909165.1_ASM190916v1	L11
GCF_000783615.1_ASM78361v2	L12
GCF_000738535.1_ASM73853v1	L13
GCF_000783975.2_ASM78397v2	L14
GCF_000465615.2_GS_De_Novo_Assembly	L15
GCF_001902635.1_ASM190263v1	L16

Supplementary Table 2. cgSNP-distance matrix of outbreak strains and reference strains created using snp-dists 0.7.0 from the parsnp coregenome alignment and P9a as reference.

snp-dists 0.7.0	B1a	rL14	P51	P11	Sc3a	P12	P41	rL9b	P55	P31a	P52	P24	Si5	P13	P57	P17	P33	P34	P56	L15	P8b	rL12	P14	P6a	P28
B1a	0	20643	3721	4147	3716	2	5419	3955	3719	5419	3717	20986	3721	18876	3717	18876	5419	5419	3717	18846	18877	14102	18876	18876	5419
B1b	3566	20643	3841	3935	3838	3565	5263	3694	3839	5263	3837	21000	3841	18816	3837	18816	5263	5263	3837	18835	18817	14234	18816	18816	5263
L9a	3514	20855	3844	4022	3841	3512	5561	3759	3842	5561	3840	21154	3844	18966	3840	18966	5561	5561	3840	18920	18967	14045	18966	18966	5561
L9b	3955	20610	4033	3765	4030	3954	5371	0	4031	5371	4029	20954	4033	18836	4029	18836	5371	5371	4029	18792	18837	14151	18836	18836	5371
P10	18876	18060	18806	18819	18801	18879	18808	18836	18802	18808	18802	18557	18804	0	18802	0	18808	18808	18801	18710	1	18656	0	0	18808
P11	4147	20670	3844	0	3839	4146	5361	3765	3842	5361	3840	20995	3844	18819	3840	18819	5361	5361	3840	18785	18820	14127	18819	18819	5361
P12	2	20644	3720	4146	3715	0	5418	3954	3718	5418	3716	20987	3720	18879	3716	18879	5418	5418	3716	18845	18880	14103	18879	18879	5418
P13	18876	18060	18806	18819	18801	18879	18808	18836	18802	18808	18802	18557	18804	0	18802	0	18808	18808	18801	18710	1	18656	0	0	18808
P14	18876	18060	18806	18819	18801	18879	18808	18836	18802	18808	18802	18557	18804	0	18802	0	18808	18808	18801	18710	1	18656	0	0	18808
P15	18876	18060	18806	18819	18801	18879	18808	18836	18802	18808	18802	18557	18804	0	18802	0	18808	18808	18801	18710	1	18656	0	0	18808
P16	18876	18060	18806	18819	18801	18879	18808	18836	18802	18808	18802	18557	18804	0	18802	0	18808	18808	18801	18710	1	18656	0	0	18808
P17	18876	18060	18806	18819	18801	18879	18808	18836	18802	18808	18802	18557	18804	0	18802	0	18808	18808	18801	18710	1	18656	0	0	18808
P18	18876	18060	18806	18819	18801	18879	18808	18836	18802	18808	18802	18557	18804	0	18802	0	18808	18808	18801	18710	1	18656	0	0	18808
P19	18865	17834	18780	18763	18775	18868	18773	18785	18776	18773	18776	18415	18778	4026	18776	4026	18773	18773	18776	18773	4027	18686	4026	4026	18773
P20	3566	20643	3841	3935	3838	3565	5263	3694	3839	5263	3837	21000	3841	18816	3837	18816	5263	5263	3837	18835	18817	14234	18816	18816	5263
P21	18886	17848	18755	18781	18750	18889	18765	18833	18751	18765	18751	18513	18753	4068	18751	4068	18765	18765	18751	18720	4069	18663	4068	4068	18765
P22	3503	20671	3677	3915	3673	3502	5384	3664	3675	5384	3673	21025	3677	18824	3673	18824	5384	5384	3673	18788	18825	14048	18824	18824	5384
P23	3503	20671	3677	3915	3673	3502	5384	3664	3675	5384	3673	21025	3677	18824	3673	18824	5384	5384	3673	18788	18825	14048	18824	18824	5384
P24	20986	7488	20988	20995	20982	20987	20947	20954	20985	20947	20984	0	20986	18557	20984	18557	20947	20947	20984	17328	18558	21201	18557	18557	20947
P25	20986	7488	20988	20995	20982	20987	20947	20954	20985	20947	20984	0	20986	18557	20984	18557	20947	20947	20984	17328	18558	21201	18557	18557	20947
P26	5419	20623	5371	5361	5366	5418	0	5371	5369	0	5367	20947	5371	18808	5367	18808	0	0	5367	18803	18809	14378	18808	18808	0
P27	5419	20623	5371	5361	5366	5418	0	5371	5369	0	5367	20947	5371	18808	5367	18808	0	0	5367	18803	18809	14378	18808	18808	0
P28	5419	20623	5371	5361	5366	5418	0	5371	5369	0	5367	20947	5371	18808	5367	18808	0	0	5367	18803	18809	14378	18808	18808	0
P29	5419	20623	5371	5361	5366	5418	0	5371	5369	0	5367	20947	5371	18808	5367	18808	0	0	5367	18803	18809	14378	18808	18808	0
P3	18876	18060	18806	18819	18801	18879	18808	18836	18802	18808	18802	18557	18804	0	18802	0	18808	18808	18801	18710	1	18656	0	0	18808
P30	5419	20623	5371	5361	5366	5418	0	5371	5369	0	5367	20947	5371	18808	5367	18808	0	0	5367	18803	18809	14378	18808	18808	0
P31a	5419	20623	5371	5361	5366	5418	0	5371	5369	0	5367	20947	5371	18808	5367	18808	0	0	5367	18803	18809	14378	18808	18808	0
P31b	5419	20623	5371	5361	5366	5418	0	5371	5369	0	5367	20947	5371	18808	5367	18808	0	0	5367	18803	18809	14378	18808	18808	0
P32	5419	20623	5371	5361	5366	5418	0	5371	5369	0	5367	20947	5371	18808	5367	18808	0	0	5367	18803	18809	14378	18808	18808	0
P33	5419	20623	5371	5361	5366	5418	0	5371	5369	0	5367	20947	5371	18808	5367	18808	0	0	5367	18803	18809	14378	18808	18808	0
P34	5419	20623	5371	5361	5366	5418	0	5371	5369	0	5367	20947	5371	18808	5367	18808	0	0	5367	18803	18809	14378	18808	18808	0
P35	5419	20623	5371	5361	5366	5418	0	5371	5369	0	5367	20947	5371	18808	5367	18808	0	0	5367	18803	18809	14378	18808	18808	0
P36	5419	20623	5371	5361	5366	5418	0	5371	5369	0	5367	20947	5371	18808	5367	18808	0	0	5367	18803	18809	14378	18808	18808	0
P37	5419	20623	5371	5361	5366	5418	0	5371	5369	0	5367	20947	5371	18808	5367	18808	0	0	5367	18803	18809	14378	18808	18808	0
P38	5419	20623	5371	5361	5366	5418	0	5371	5369	0	5367	20947	5371	18808	5367	18808	0	0	5367	18803	18809	14378	18808	18808	0
P39	5419	20623	5371	5361	5366	5418	0	5371	5369	0	5367	20947	5371	18808	5367	18808	0	0	5367	18803	18809	14378	18808	18808	0
P41	5419	20623	5371	5361	5366	5418	0	5371	5369	0	5367	20947	5371	18808	5367	18808	0	0	5367	18803	18809	14378	18808	18808	0
P42	5419	20623	5371	5361	5366	5418	0	5371	5369	0	5367	20947	5371	18808	5367	18808	0	0	5367	18803	18809	14378	18808	18808	0
P43	5419	20623	5371	5361	5366	5418	0	5371	5369	0	5367	20947	5371	18808	5367	18808	0	0	5367	18803	18809	14378	18808	18808	0
P44	5419	20623	5371	5361	5366	5418	0	5371	5369	0	5367	20947	5371	18808	5367	18808	0	0	5367	18803	18809	14378	18808	18808	0
P46	5419	20623	5371	5361	5366	5418	0	5371	5369	0	5367	20947	5371	18808	5367	18808	0	0	5367	18803	18809	14378	18808	18808	0
P47	5419	20623	5371	5361	5366	5418	0	5371	5369	0	5367	20947	5371	18808	5367	18808	0	0	5367	18803	18809	14378	18808	18808	0
P48	18596	18132	18536	18524	18532	18595	18569	18608	18534	18569	18532	18555	18536	17382	18532	17382	18569	18569	18531	12994	17383	19191	17382	17382	18569
P49	18596	18132	18536	18524	18532	18595	18569	18608	18534	18569	18532	18555	18536	17382	18532	17382	18569	18569	18531	12994	17383	19191	17382	17382	18569
P5	18876	18060	18806	18819	18801	18879	18808	18836	18802	18808	18802	18557	18804	0	18802	0	18808	18808	18801	18710	1	18656	0	0	18808
P50	3718	20649	5	3841	2	3717	5368	4030	3	5368	1	20985	5	18803	1	18803	5368	5368	1	18761	18804	14061	18803	18803	5368
P51	3721	20652	0	3844	5	3720	5371	4033	6	5371	4	20988	8	18806	4	18806	5371	5371	4	18764	18807	14064	18806	18806	5371
P52	3717	20648	4	3840	1	3716	5367	4029	2	5367	0	20984	4	18802	0	18802	5367	5367	0	18760	18803	14060	18802	18802	5367
P53	3718	20649	5	3841	2	3717	5368	4030	3	5368	1	20985	5	18803	1	18803	5368	5368	1	18761	18804	14061	18803	18803	5368
P54	3717	20648	4	3839	1	3716	5367	4029	2	5367	0	20984	4	18802	0	18802	5367	5367	0	18760	18803	14060	18802	18802	5367
P55	3719	20649	6	3842	3	3718	5369	4031	0	5369	2	20985	6	18802	2	18802	5369	5369	2	18762	18803	14061	18802	18802	5369
P56	3717	20648	4	3840	1	3716	5367	4029	2	5367	0	20984	4	18801	0	18801	5367	5367	0	18760	18802	14060	18801	18801	5367
P57	3717	20648	4	3840	1	3716	5367	4029	2	5367	0	20984	4	18802	0	18802	5367	5367	0	18760	18803	14060	18802	18802	5367
P58	18406	18208	18318	18314	18314	18405	18361	18386	18316	18361	18314	18602	18318	17097	18314	17097	18361	18361	18313	12919	17098	18974	17097	17097	18361
P6a	18876	18060	18806	18819	18801	18879	18808	18836	18802	18808	18802	18557	18804	0	18802	0	18808	18808	18801	18710	1	18656	0	0	18808
P6b	18876	18060	18806	18819	18801	18879	18808	18836	18802	18808	18802	18557	18804	0	18802	0	18808	18808	18801	18710	1	18656	0	0	18808
P7	18876	18060	18806	18819	18801	18879	18808	18836	18802	18808	18802	18557	18804	0	18802	0	18808	18808	18801	18710	1	18656	0	0	18808
P8a	18876	18060	18806	18819	18801	18879	18808	18836	18802	18808	18802	18557	18804	0	18802	0	18808	18808	18801	18710	1	18656	0	0	18808
P8b	18877	18061	18807	18820	18802	18880	18809	18837	18803	18809	18803	18558	18805	1	18803	1	18809	18809	18802	18711	0	18657	1	1	18809
P9	2	20644	3720	4146	3715	0	5418	3954	3718	5418	3716	20987	3720	18879	3716	18879	5418	5418	3716	18845	18880	14103	18879	18879	5418
rL10	22058	22577	21967	22020	21959	22059	22053	21984	21962	22053	21962	22075	21967	23081	21963	23081	22053	22053	21963	20517	23082	21259	23081	23081	22053
rL11	21653	20371	21610	21629	21602	21654	21685	21625	21607	21685	21605	19715	21610	22215	21606	22215	21685	21685	21606	18982	22216	20894	22215	22215	21685
rL12	14102	20674	14064	14127	14059	14103	14378	14151	14061	14378	14060	21201	14064	18656	14060	18656	14378	14378	14060	19182	18657	0	18656	18656	14378
rL13	18859	17815	18753	18759	18747	18862	18747	18810	18749	18747	18749	18360	18751	4023	18749	4023	18747	18747	18749	18705	4024	18624	4023	4023	18747
rL14	20643	0	20652	20670	20647	20644	20623	20610	20649	20623	20648	7488	20650	18060	20648	18060	20623	20623	20648	17641	18061	20674	18060	18060	20623
rL15	18846	17641	18764	18785	18758	18845	18803	18792	18762	18803	18760	17328	18762	18710	18760	18710	18803	18803	18760	0	18711	19182	18710	18710	18803
Sc1a	3566	20643	3841	3935	3838	3565	5263	3694	3839	5263	3837	21000	3841	18816	3837	18816	5263	5263	3837	18835	18817	14234	18816	18816	5263
Sc1b	3717	20648	4	3840	1	3716	5367	4029	2	5367	0	20984	4	18802	0	18802	5367	5367	0	18760	18803	14060	18802	18802	5367
Sc2	3917	20727	4123	4311	4120	3916	5688	4220	4121	5688	4119	21002	4123	18883	4119	18883	5688	5688	4119	18845	18884	14084	18883	18883	5688
Sc3a	3716	20647	5	3839	0	3715	5366	4030	3	5366	1	20982	5	18801	1	18801	5366	5366	1	18758	18802	14059	18801	18801	5366
Sc3b	5419	20623	5371	5361	5366	5418	0	5371	5369	0	5367	20947	5371	18808	5367	18808	0	0	5367	18803	18809	14378	18808	18808	0
Si5	3721	20650	8	3844	5	3720	5371	4033	6	5371	4	20986	0	18804	4	18804	5371	5371	4	18762	18805	14064	18804	18804	5371

snp-dists 0.7.0	P50	P53	P38	P35	P26	P49	P42	rL11	P58	Sc1a	P16	P21	Sc1b	P15	Sc2	B1b	P37	P30	Sc3b	P31b	P39	P25	P9	P48	P22
B1a	3718	3718	5419	5419	5419	18596	5419	21653	18406	3566	18876	18886	3717	18876	3917	3566	5419	5419	5419	5419	5419	20986	2	18596	3503
B1b	3838	3838	5263	5263	5263	18592	5263	21676	18381	0	18816	18798	3837	18816	4079	0	5263	5263	5263	5263	5263	21000	3565	18592	3445
L9a	3841	3841	5561	5561	5561	18660	5561	21716	18471	3505	18966	18952	3840	18966	3866	3505	5561	5561	5561	5561	5561	21154	3512	18660	2503
L9b	4030	4030	5371	5371	5371	18608	5371	21625	18386	3694	18836	18833	4029	18836	4220	3694	5371	5371	5371	5371	5371	20954	3954	18608	3664
P10	18803	18803	18808	18808	18808	17382	18808	22215	17097	18816	0	4068	18802	0	18883	18816	18808	18808	18808	18808	18808	18557	18879	17382	18824
P11	3841	3841	5361	5361	5361	18524	5361	21629	18314	3935	18819	18781	3840	18819	4311	3935	5361	5361	5361	5361	5361	20995	4146	18524	3915
P12	3717	3717	5418	5418	5418	18595	5418	21654	18405	3565	18879	18889	3716	18879	3916	3565	5418	5418	5418	5418	5418	20987	0	18595	3502
P13	18803	18803	18808	18808	18808	17382	18808	22215	17097	18816	0	4068	18802	0	18883	18816	18808	18808	18808	18808	18808	18557	18879	17382	18824
P14	18803	18803	18808	18808	18808	17382	18808	22215	17097	18816	0	4068	18802	0	18883	18816	18808	18808	18808	18808	18808	18557	18879	17382	18824
P15	18803	18803	18808	18808	18808	17382	18808	22215	17097	18816	0	4068	18802	0	18883	18816	18808	18808	18808	18808	18808	18557	18879	17382	18824
P16	18803	18803	18808	18808	18808	17382	18808	22215	17097	18816	0	4068	18802	0	18883	18816	18808	18808	18808	18808	18808	18557	18879	17382	18824
P17	18803	18803	18808	18808	18808	17382	18808	22215	17097	18816	0	4068	18802	0	18883	18816	18808	18808	18808	18808	18808	18557	18879	17382	18824
P18	18803	18803	18808	18808	18808	17382	18808	22215	17097	18816	0	4068	18802	0	18883	18816	18808	18808	18808	18808	18808	18557	18879	17382	18824
P19	18777	18777	18773	18773	18773	17367	18773	22133	17044	18799	4026	3856	18776	4026	18830	18799	18773	18773	18773	18773	18773	18415	18868	17367	18792
P20	3838	3838	5263	5263	5263	18592	5263	21676	18381	0	18816	18798	3837	18816	4079	0	5263	5263	5263	5263	5263	21000	3565	18592	3445
P21	18752	18752	18765	18765	18765	17361	18765	22210	17117	18798	4068	0	18751	4068	18858	18798	18765	18765	18765	18765	18765	18513	18889	17361	18807
P22	3674	3674	5384	5384	5384	18580	5384	21573	18358	3445	18824	18807	3673	18824	3845	3445	5384	5384	5384	5384	5384	21025	3502	18580	0
P23	3674	3674	5384	5384	5384	18580	5384	21573	18358	3445	18824	18807	3673	18824	3845	3445	5384	5384	5384	5384	5384	21025	3502	18580	0
P24	20985	20985	20947	20947	20947	18555	20947	19715	18602	21000	18557	18513	20984	18557	21002	21000	20947	20947	20947	20947	20947	0	20987	18555	21025
P25	20985	20985	20947	20947	20947	18555	20947	19715	18602	21000	18557	18513	20984	18557	21002	21000	20947	20947	20947	20947	20947	0	20987	18555	21025
P26	5368	5368	0	0	0	18569	0	21685	18361	5263	18808	18765	5367	18808	5688	5263	0	0	0	0	0	20947	5418	18569	5384
P27	5368	5368	0	0	0	18569	0	21685	18361	5263	18808	18765	5367	18808	5688	5263	0	0	0	0	0	20947	5418	18569	5384
P28	5368	5368	0	0	0	18569	0	21685	18361	5263	18808	18765	5367	18808	5688	5263	0	0	0	0	0	20947	5418	18569	5384
P29	5368	5368	0	0	0	18569	0	21685	18361	5263	18808	18765	5367	18808	5688	5263	0	0	0	0	0	20947	5418	18569	5384
P3	18803	18803	18808	18808	18808	17382	18808	22215	17097	18816	0	4068	18802	0	18883	18816	18808	18808	18808	18808	18808	18557	18879	17382	18824
P30	5368	5368	0	0	0	18569	0	21685	18361	5263	18808	18765	5367	18808	5688	5263	0	0	0	0	0	20947	5418	18569	5384
P31a	5368	5368	0	0	0	18569	0	21685	18361	5263	18808	18765	5367	18808	5688	5263	0	0	0	0	0	20947	5418	18569	5384
P31b	5368	5368	0	0	0	18569	0	21685	18361	5263	18808	18765	5367	18808	5688	5263	0	0	0	0	0	20947	5418	18569	5384
P32	5368	5368	0	0	0	18569	0	21685	18361	5263	18808	18765	5367	18808	5688	5263	0	0	0	0	0	20947	5418	18569	5384
P33	5368	5368	0	0	0	18569	0	21685	18361	5263	18808	18765	5367	18808	5688	5263	0	0	0	0	0	20947	5418	18569	5384
P34	5368	5368	0	0	0	18569	0	21685	18361	5263	18808	18765	5367	18808	5688	5263	0	0	0	0	0	20947	5418	18569	5384
P35	5368	5368	0	0	0	18569	0	21685	18361	5263	18808	18765	5367	18808	5688	5263	0	0	0	0	0	20947	5418	18569	5384
P36	5368	5368	0	0	0	18569	0	21685	18361	5263	18808	18765	5367	18808	5688	5263	0	0	0	0	0	20947	5418	18569	5384
P37	5368	5368	0	0	0	18569	0	21685	18361	5263	18808	18765	5367	18808	5688	5263	0	0	0	0	0	20947	5418	18569	5384
P38	5368	5368	0	0	0	18569	0	21685	18361	5263	18808	18765	5367	18808	5688	5263	0	0	0	0	0	20947	5418	18569	5384
P39	5368	5368	0	0	0	18569	0	21685	18361	5263	18808	18765	5367	18808	5688	5263	0	0	0	0	0	20947	5418	18569	5384
P41	5368	5368	0	0	0	18569	0	21685	18361	5263	18808	18765	5367	18808	5688	5263	0	0	0	0	0	20947	5418	18569	5384
P42	5368	5368	0	0	0	18569	0	21685	18361	5263	18808	18765	5367	18808	5688	5263	0	0	0	0	0	20947	5418	18569	5384
P43	5368	5368	0	0	0	18569	0	21685	18361	5263	18808	18765	5367	18808	5688	5263	0	0	0	0	0	20947	5418	18569	5384
P44	5368	5368	0	0	0	18569	0	21685	18361	5263	18808	18765	5367	18808	5688	5263	0	0	0	0	0	20947	5418	18569	5384
P46	5368	5368	0	0	0	18569	0	21685	18361	5263	18808	18765	5367	18808	5688	5263	0	0	0	0	0	20947	5418	18569	5384
P47	5368	5368	0	0	0	18569	0	21685	18361	5263	18808	18765	5367	18808	5688	5263	0	0	0	0	0	20947	5418	18569	5384
P48	18533	18533	18569	18569	18569	0	18569	21246	4711	18592	17382	17361	18532	17382	18611	18592	18569	18569	18569	18569	18569	18555	18595	0	18580
P49	18533	18533	18569	18569	18569	0	18569	21246	4711	18592	17382	17361	18532	17382	18611	18592	18569	18569	18569	18569	18569	18555	18595	0	18580
P5	18803	18803	18808	18808	18808	17382	18808	22215	17097	18816	0	4068	18802	0	18883	18816	18808	18808	18808	18808	18808	18557	18879	17382	18824
P50	0	2	5368	5368	5368	18533	5368	21607	18315	3838	18803	18752	1	18803	4120	3838	5368	5368	5368	5368	5368	20985	3717	18533	3674
P51	5	5	5371	5371	5371	18536	5371	21610	18318	3841	18806	18755	4	18806	4123	3841	5371	5371	5371	5371	5371	20988	3720	18536	3677
P52	1	1	5367	5367	5367	18532	5367	21605	18314	3837	18802	18751	0	18802	4119	3837	5367	5367	5367	5367	5367	20984	3716	18532	3673
P53	2	0	5368	5368	5368	18533	5368	21607	18315	3838	18803	18752	1	18803	4120	3838	5368	5368	5368	5368	5368	20985	3717	18533	3674
P54	1	1	5367	5367	5367	18532	5367	21605	18314	3837	18802	18751	0	18802	4119	3837	5367	5367	5367	5367	5367	20984	3716	18532	3673
P55	3	3	5369	5369	5369	18534	5369	21607	18316	3839	18802	18751	2	18802	4121	3839	5369	5369	5369	5369	5369	20985	3718	18534	3675
P56	1	1	5367	5367	5367	18531	5367	21606	18313	3837	18801	18751	0	18801	4119	3837	5367	5367	5367	5367	5367	20984	3716	18531	3673
P57	1	1	5367	5367	5367	18532	5367	21606	18314	3837	18802	18751	0	18802	4119	3837	5367	5367	5367	5367	5367	20984	3716	18532	3673
P58	18315	18315	18361	18361	18361	4711	18361	21153	0	18381	17097	17117	18314	17097	18390	18381	18361	18361	18361	18361	18361	18602	18405	4711	18358
P6a	18803	18803	18808	18808	18808	17382	18808	22215	17097	18816	0	4068	18802	0	18883	18816	18808	18808	18808	18808	18808	18557	18879	17382	18824
P6b	18803	18803	18808	18808	18808	17382	18808	22215	17097	18816	0	4068	18802	0	18883	18816	18808	18808	18808	18808	18808	18557	18879	17382	18824
P7	18803	18803	18808	18808	18808	17382	18808	22215	17097	18816	0	4068	18802	0	18883	18816	18808	18808	18808	18808	18808	18557	18879	17382	18824
P8a	18803	18803	18808	18808	18808	17382	18808	22215	17097	18816	0	4068	18802	0	18883	18816	18808	18808	18808	18808	18808	18557	18879	17382	18824
P8b	18804	18804	18809	18809	18809	17383	18809	22216	17098	18817	1	4069	18803	1	18884	18817	18809	18809	18809	18809	18809	18558	18880	17383	18825
P9	3717	3717	5418	5418	5418	18595	5418	21654	18405	3565	18879	18889	3716	18879	3916	3565	5418	5418	5418	5418	5418	20987	0	18595	3502
rL10	21964	21964	22053	22053	22053	22443	22053	8013	22269	22043	23081	23083	21963	23081	22082	22043	22053	22053	22053	22053	22053	22075	22059	22443	21998
rL11	21607	21607	21685	21685	21685	21246	21685	0	21153	21676	22215	22210	21606	22215	21682	21676	21685	21685	21685	21685	21685	19715	21654	21246	21573
rL12	14061	14061	14378	14378	14378	19191	14378	20894	18974	14234	18656	18663	14060	18656	14084	14234	14378	14378	14378	14378	14378	21201	14103	19191	14048
rL13	18750	18750	18747	18747	18747	17294	18747	22235	17074	18788	4023	3705	18749	4023	18834	18788	18747	18747	18747	18747	18747	18360	18862	17294	18811
rL14	20649	20649	20623	20623	20623	18132	20623	20371	18208	20643	18060	17848	20648	18060	20727	20643	20623	20623	20623	20623	20623	7488	20644	18132	20671
rL15	18761	18761	18803	18803	18803	12994	18803	18982	12919	18835	18710	18720	18760	18710	18845	18835	18803	18803	18803	18803	18803	17328	18845	12994	18788
Sc1a	3838	3838	5263	5263	5263	18592	5263	21676	18381	0	18816	18798	3837	18816	4079	0	5263	5263	5263	5263	5263	21000	3565	18592	3445
Sc1b	1	1	5367	5367	5367	18532	5367	21606	18314	3837	18802	18751	0	18802	4119	3837	5367	5367	5367	5367	5367	20984	3716	18532	3673
Sc2	4120	4120	5688	5688	5688	18611	5688	21682	18390	4079	18883	18858	4119	18883	0	4079	5688	5688	5688	5688	5688	21002	3916	18611	3845
Sc3a	2	2	5366	5366	5366	18532	5366	21602	18314	3838	18801	18750	1	18801	4120	3838	5366	5366	5366	5366	5366	20982	3715	18532	3673
Sc3b	5368	5368	0	0	0	18569	0	21685	18361	5263	18808	18765	5367	18808	5688	5263	0	0	0	0	0	20947	5418	18569	5384
Si5	5	5	5371	5371	5371	18536	5371	21610	18318	3841	18804	18753	4	18804	4123	3841	5371	5371	5371	5371	5371	20986	3720	18536	3677

snp-dists 0.7.0	P46	P20	P27	P29	P6b	rL13	P5	P54	P18	P7	P36	P32	P19	rL9a	P8a	P3	P44	P23	P43	P47	P10	rL10
B1a	5419	3566	5419	5419	18876	18859	18876	3717	18876	18876	5419	5419	18865	3514	18876	18876	5419	3503	5419	5419	18876	22058
B1b	5263	0	5263	5263	18816	18788	18816	3837	18816	18816	5263	5263	18799	3505	18816	18816	5263	3445	5263	5263	18816	22043
L9a	5561	3505	5561	5561	18966	18949	18966	3840	18966	18966	5561	5561	18940	0	18966	18966	5561	2503	5561	5561	18966	22148
L9b	5371	3694	5371	5371	18836	18810	18836	4029	18836	18836	5371	5371	18785	3759	18836	18836	5371	3664	5371	5371	18836	21984
P10	18808	18816	18808	18808	0	4023	0	18802	0	0	18808	18808	4026	18966	0	0	18808	18824	18808	18808	0	23081
P11	5361	3935	5361	5361	18819	18759	18819	3839	18819	18819	5361	5361	18763	4022	18819	18819	5361	3915	5361	5361	18819	22020
P12	5418	3565	5418	5418	18879	18862	18879	3716	18879	18879	5418	5418	18868	3512	18879	18879	5418	3502	5418	5418	18879	22059
P13	18808	18816	18808	18808	0	4023	0	18802	0	0	18808	18808	4026	18966	0	0	18808	18824	18808	18808	0	23081
P14	18808	18816	18808	18808	0	4023	0	18802	0	0	18808	18808	4026	18966	0	0	18808	18824	18808	18808	0	23081
P15	18808	18816	18808	18808	0	4023	0	18802	0	0	18808	18808	4026	18966	0	0	18808	18824	18808	18808	0	23081
P16	18808	18816	18808	18808	0	4023	0	18802	0	0	18808	18808	4026	18966	0	0	18808	18824	18808	18808	0	23081
P17	18808	18816	18808	18808	0	4023	0	18802	0	0	18808	18808	4026	18966	0	0	18808	18824	18808	18808	0	23081
P18	18808	18816	18808	18808	0	4023	0	18802	0	0	18808	18808	4026	18966	0	0	18808	18824	18808	18808	0	23081
P19	18773	18799	18773	18773	4026	3830	4026	18776	4026	4026	18773	18773	0	18940	4026	4026	18773	18792	18773	18773	4026	23031
P20	5263	0	5263	5263	18816	18788	18816	3837	18816	18816	5263	5263	18799	3505	18816	18816	5263	3445	5263	5263	18816	22043
P21	18765	18798	18765	18765	4068	3705	4068	18751	4068	4068	18765	18765	3856	18952	4068	4068	18765	18807	18765	18765	4068	23083
P22	5384	3445	5384	5384	18824	18811	18824	3673	18824	18824	5384	5384	18792	2503	18824	18824	5384	0	5384	5384	18824	21998
P23	5384	3445	5384	5384	18824	18811	18824	3673	18824	18824	5384	5384	18792	2503	18824	18824	5384	0	5384	5384	18824	21998
P24	20947	21000	20947	20947	18557	18360	18557	20984	18557	18557	20947	20947	18415	21154	18557	18557	20947	21025	20947	20947	18557	22075
P25	20947	21000	20947	20947	18557	18360	18557	20984	18557	18557	20947	20947	18415	21154	18557	18557	20947	21025	20947	20947	18557	22075
P26	0	5263	0	0	18808	18747	18808	5367	18808	18808	0	0	18773	5561	18808	18808	0	5384	0	0	18808	22053
P27	0	5263	0	0	18808	18747	18808	5367	18808	18808	0	0	18773	5561	18808	18808	0	5384	0	0	18808	22053
P28	0	5263	0	0	18808	18747	18808	5367	18808	18808	0	0	18773	5561	18808	18808	0	5384	0	0	18808	22053
P29	0	5263	0	0	18808	18747	18808	5367	18808	18808	0	0	18773	5561	18808	18808	0	5384	0	0	18808	22053
P3	18808	18816	18808	18808	0	4023	0	18802	0	0	18808	18808	4026	18966	0	0	18808	18824	18808	18808	0	23081
P30	0	5263	0	0	18808	18747	18808	5367	18808	18808	0	0	18773	5561	18808	18808	0	5384	0	0	18808	22053
P31a	0	5263	0	0	18808	18747	18808	5367	18808	18808	0	0	18773	5561	18808	18808	0	5384	0	0	18808	22053
P31b	0	5263	0	0	18808	18747	18808	5367	18808	18808	0	0	18773	5561	18808	18808	0	5384	0	0	18808	22053
P32	0	5263	0	0	18808	18747	18808	5367	18808	18808	0	0	18773	5561	18808	18808	0	5384	0	0	18808	22053
P33	0	5263	0	0	18808	18747	18808	5367	18808	18808	0	0	18773	5561	18808	18808	0	5384	0	0	18808	22053
P34	0	5263	0	0	18808	18747	18808	5367	18808	18808	0	0	18773	5561	18808	18808	0	5384	0	0	18808	22053
P35	0	5263	0	0	18808	18747	18808	5367	18808	18808	0	0	18773	5561	18808	18808	0	5384	0	0	18808	22053
P36	0	5263	0	0	18808	18747	18808	5367	18808	18808	0	0	18773	5561	18808	18808	0	5384	0	0	18808	22053
P37	0	5263	0	0	18808	18747	18808	5367	18808	18808	0	0	18773	5561	18808	18808	0	5384	0	0	18808	22053
P38	0	5263	0	0	18808	18747	18808	5367	18808	18808	0	0	18773	5561	18808	18808	0	5384	0	0	18808	22053
P39	0	5263	0	0	18808	18747	18808	5367	18808	18808	0	0	18773	5561	18808	18808	0	5384	0	0	18808	22053
P41	0	5263	0	0	18808	18747	18808	5367	18808	18808	0	0	18773	5561	18808	18808	0	5384	0	0	18808	22053
P42	0	5263	0	0	18808	18747	18808	5367	18808	18808	0	0	18773	5561	18808	18808	0	5384	0	0	18808	22053
P43	0	5263	0	0	18808	18747	18808	5367	18808	18808	0	0	18773	5561	18808	18808	0	5384	0	0	18808	22053
P44	0	5263	0	0	18808	18747	18808	5367	18808	18808	0	0	18773	5561	18808	18808	0	5384	0	0	18808	22053
P46	0	5263	0	0	18808	18747	18808	5367	18808	18808	0	0	18773	5561	18808	18808	0	5384	0	0	18808	22053
P47	0	5263	0	0	18808	18747	18808	5367	18808	18808	0	0	18773	5561	18808	18808	0	5384	0	0	18808	22053
P48	18569	18592	18569	18569	17382	17294	17382	18532	17382	17382	18569	18569	17367	18660	17382	17382	18569	18580	18569	18569	17382	22443
P49	18569	18592	18569	18569	17382	17294	17382	18532	17382	17382	18569	18569	17367	18660	17382	17382	18569	18580	18569	18569	17382	22443
P5	18808	18816	18808	18808	0	4023	0	18802	0	0	18808	18808	4026	18966	0	0	18808	18824	18808	18808	0	23081
P50	5368	3838	5368	5368	18803	18750	18803	1	18803	18803	5368	5368	18777	3841	18803	18803	5368	3674	5368	5368	18803	21964
P51	5371	3841	5371	5371	18806	18753	18806	4	18806	18806	5371	5371	18780	3844	18806	18806	5371	3677	5371	5371	18806	21967
P52	5367	3837	5367	5367	18802	18749	18802	0	18802	18802	5367	5367	18776	3840	18802	18802	5367	3673	5367	5367	18802	21962
P53	5368	3838	5368	5368	18803	18750	18803	1	18803	18803	5368	5368	18777	3841	18803	18803	5368	3674	5368	5368	18803	21964
P54	5367	3837	5367	5367	18802	18749	18802	0	18802	18802	5367	5367	18776	3840	18802	18802	5367	3673	5367	5367	18802	21963
P55	5369	3839	5369	5369	18802	18749	18802	2	18802	18802	5369	5369	18776	3842	18802	18802	5369	3675	5369	5369	18802	21962
P56	5367	3837	5367	5367	18801	18749	18801	0	18801	18801	5367	5367	18776	3840	18801	18801	5367	3673	5367	5367	18801	21963
P57	5367	3837	5367	5367	18802	18749	18802	0	18802	18802	5367	5367	18776	3840	18802	18802	5367	3673	5367	5367	18802	21963
P58	18361	18381	18361	18361	17097	17074	17097	18314	17097	17097	18361	18361	17044	18471	17097	17097	18361	18358	18361	18361	17097	22269
P6a	18808	18816	18808	18808	0	4023	0	18802	0	0	18808	18808	4026	18966	0	0	18808	18824	18808	18808	0	23081
P6b	18808	18816	18808	18808	0	4023	0	18802	0	0	18808	18808	4026	18966	0	0	18808	18824	18808	18808	0	23081
P7	18808	18816	18808	18808	0	4023	0	18802	0	0	18808	18808	4026	18966	0	0	18808	18824	18808	18808	0	23081
P8a	18808	18816	18808	18808	0	4023	0	18802	0	0	18808	18808	4026	18966	0	0	18808	18824	18808	18808	0	23081
P8b	18809	18817	18809	18809	1	4024	1	18803	1	1	18809	18809	4027	18967	1	1	18809	18825	18809	18809	1	23082
P9	5418	3565	5418	5418	18879	18862	18879	3716	18879	18879	5418	5418	18868	3512	18879	18879	5418	3502	5418	5418	18879	22059
rL10	22053	22043	22053	22053	23081	23101	23081	21963	23081	23081	22053	22053	23031	22148	23081	23081	22053	21998	22053	22053	23081	0
rL11	21685	21676	21685	21685	22215	22235	22215	21605	22215	22215	21685	21685	22133	21716	22215	22215	21685	21573	21685	21685	22215	8013
rL12	14378	14234	14378	14378	18656	18624	18656	14060	18656	18656	14378	14378	18686	14045	18656	18656	14378	14048	14378	14378	18656	21259
rL13	18747	18788	18747	18747	4023	0	4023	18749	4023	4023	18747	18747	3830	18949	4023	4023	18747	18811	18747	18747	4023	23101
rL14	20623	20643	20623	20623	18060	17815	18060	20648	18060	18060	20623	20623	17834	20855	18060	18060	20623	20671	20623	20623	18060	22577
rL15	18803	18835	18803	18803	18710	18705	18710	18760	18710	18710	18803	18803	18773	18920	18710	18710	18803	18788	18803	18803	18710	20517
Sc1a	5263	0	5263	5263	18816	18788	18816	3837	18816	18816	5263	5263	18799	3505	18816	18816	5263	3445	5263	5263	18816	22043
Sc1b	5367	3837	5367	5367	18802	18749	18802	0	18802	18802	5367	5367	18776	3840	18802	18802	5367	3673	5367	5367	18802	21963
Sc2	5688	4079	5688	5688	18883	18834	18883	4119	18883	18883	5688	5688	18830	3866	18883	18883	5688	3845	5688	5688	18883	22082
Sc3a	5366	3838	5366	5366	18801	18747	18801	1	18801	18801	5366	5366	18775	3841	18801	18801	5366	3673	5366	5366	18801	21959
Sc3b	0	5263	0	0	18808	18747	18808	5367	18808	18808	0	0	18773	5561	18808	18808	0	5384	0	0	18808	22053
Si5	5371	3841	5371	5371	18804	18751	18804	4	18804	18804	5371	5371	18778	3844	18804	18804	5371	3677	5371	5371	18804	21967

Supplementary Figure 1. Schematic representation of the NICU floor plan. B: bath; Sc: scrub; Si: washbasin; N1: intensive care area; N2: medium care area.

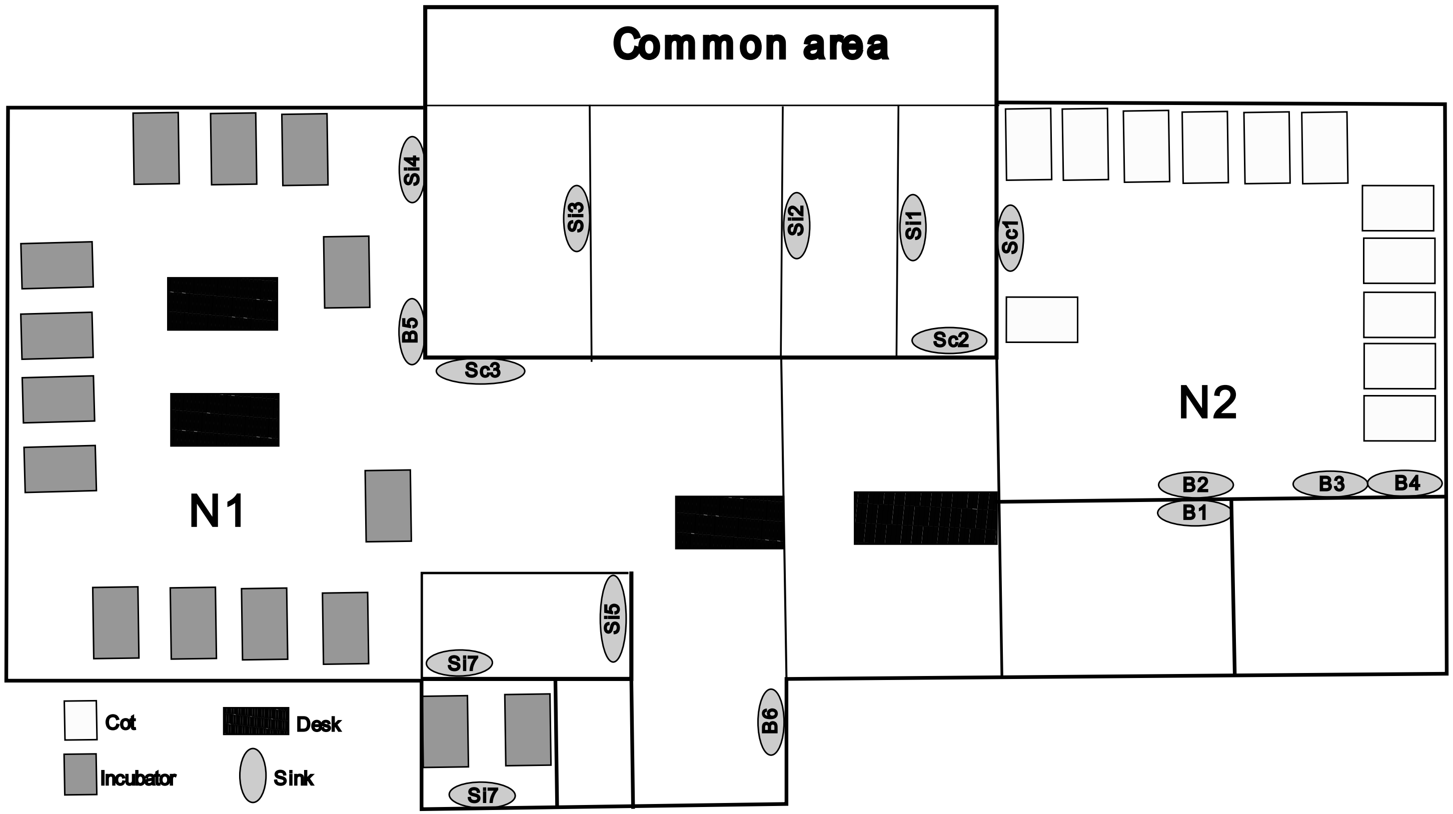

Supplement: Supplementary Material [file 24-00144_MALHOTRA-KUMAR_Supplement.pdf]
